# Transcriptional Regulation Analysis Provides Insight into the Function of *GSK3β* Gene in Diannan Small-Ear Pig Spermatogenesis

**DOI:** 10.3390/genes15060655

**Published:** 2024-05-22

**Authors:** Xia Zhang, Guiying Zhao, Fuhua Yang, Changyao Li, Wan Lin, Hongmei Dai, Lan Zhai, Xuemin Xi, Qingting Yuan, Jinlong Huo

**Affiliations:** 1 Department of Biological and Food Engineering, Lyuliang University, Lvliang 033001, China; xiazhang1425@163.com; 2 College of Animal Science and Technology, Yunnan Agricultural University, Kunming 650201, China; zhaoguiying2006@163.com (G.Z.); y18213377954@outlook.com (F.Y.); changyaoli01@163.com (C.L.); wanlin2518@163.com (W.L.); hongmeidai9796@163.com (H.D.); zhailan3544@163.com (L.Z.); xueminxi0111@163.com (X.X.); qingtingyuan0523@163.com (Q.Y.)

**Keywords:** Diannan small-ear (DSE) pigs, glycogen synthase kinase-3 beta (*GSK3β*), long- and short-read sequencing, transcriptional regulation

## Abstract

Glycogen synthase kinase-3β (GSK3β) not only plays a crucial role in regulating sperm maturation but also is pivotal in orchestrating the acrosome reaction. Here, we integrated single-molecule long-read and short-read sequencing to comprehensively examine *GSK3β* expression patterns in adult Diannan small-ear pig (DSE) testes. We identified the most important transcript ENSSSCT00000039364 of *GSK3β*, obtaining its full-length coding sequence (CDS) spanning 1263 bp. Gene structure analysis located *GSK3β* on pig chromosome 13 with 12 exons. Protein structure analysis reflected that *GSK3β* consisted of 420 amino acids containing PKc-like conserved domains. Phylogenetic analysis underscored the evolutionary conservation and homology of *GSK3β* across different mammalian species. The evaluation of the protein interaction network, KEGG, and GO pathways implied that GSK3β interacted with 50 proteins, predominantly involved in the Wnt signaling pathway, papillomavirus infection, hippo signaling pathway, hepatocellular carcinoma, gastric cancer, colorectal cancer, breast cancer, endometrial cancer, basal cell carcinoma, and Alzheimer’s disease. Functional annotation identified that *GSK3β* was involved in thirteen GOs, including six molecular functions and seven biological processes. ceRNA network analysis suggested that DSE *GSK3β* was regulated by 11 miRNA targets. Furthermore, qPCR expression analysis across 15 tissues highlighted that *GSK3β* was highly expressed in the testis. Subcellular localization analysis indicated that the majority of the GSK3β protein was located in the cytoplasm of ST (swine testis) cells, with a small amount detected in the nucleus. Overall, our findings shed new light on *GSK3β*’s role in DSE reproduction, providing a foundation for further functional studies of *GSK3β* function.

## 1. Introduction

Spermatozoa are highly specialized cells essential for transmitting genetic information to the next generation. If mammalian sperms are immotile, the oocyte cannot be fertilized even after being released from the seminiferous tubular epithelium of the testes. Before fertilizing oocytes, sperm must undergo a series of intricate processes in the uterus and fallopian tubes. These processes include capacitation, acrosome reaction, nuclear condensation and elongation, cytoplasm elimination, and flagellar development prior to their release from the testis [1]. However, the process of overactivation and acrosome reactions is tightly regulated through the phosphorylation of specific proteins. Glycogen synthase kinase-3 (GSK3), a serine/threonine kinase with two different isoforms (α and β), has been identified in association with sperm maturation in the epididymis [2]. The levels of GSK3α/β in goat sperm increase during epididymal tail transport; the serine phosphorylation of GSK3α/β regulates goat sperm motility and acrosome response by mediating energy pathways in glycolysis and oxidative phosphorylation [3]. Notably, the catalytic activity of GSK3 is greater in immature epididymal head sperm compared to mature epididymal tail sperm. However, the pharmacological inhibition of GSK3 alone does not induce movement in quiescent sperm cells [4]. Furthermore, the GSK3α/β protein plays a crucial role not only in regulating sperm maturation in mice but also in orchestrating the acrosome reaction [4]. The absence of GSK3α in mice results in reproductive incapacity, accompanied by compromised sperm function [5].

Glycogen synthase kinase-3β (GSK3β), initially recognized as an enzyme responsible for glycogen synthase phosphorylation and deactivation, is now recognized as a ubiquitous signaling molecule pivotal in various cellular functions. GSK3β is a promising drug target for numerous neurological disorders, including Alzheimer’s disease, Parkinson’s disease, schizophrenia, and bipolar disorder, as well as conditions related to energy metabolism and cell death, such as diabetes and cancer [6,7]. The extensive use of this kinase in various diseases stems from its role as an active protein kinase that regulates nearly 40 protein substrates, all of which are modulated by signaling pathways such as Wnt, insulin, and brain-derived neurotrophic factor (BDNF). Notably, in mouse models of Alzheimer’s disease, the reduction in amyloid-β deposition and associated neuronal apoptosis during apoptosis is achieved by inhibiting GSK3β [8,9]. In the neural feedforward response mechanism, the inhibition of Wnt signaling by neurotoxic amyloid-β peptide results in the inactivation of GSK3β phosphorylation [10]. GSK3β is the primary kinase responsible for phosphorylating the microtubule-stabilizing tau protein, a key factor in the formation of insoluble paired spiral filaments and the characteristic nerve fiber tangling observed in Alzheimer’s disease [11,12]. The diverse functions carried out by GSK3β underscore its involvement in numerous cellular processes, encompassing glycogen metabolism, gene expression, proliferation, and development [13].

Integrating high-throughput short reads from second-generation sequencing (Illumina) with long reads from third-generation sequencing (PacBio) enables a more comprehensive capture of transcript full-length information, facilitating the identification of new transcripts, splice variants, and non-coding RNAs, thereby enhancing our insight into gene expression mechanisms [14]. Here, we employed Pacific Biotechnology isoform sequencing (PacBio Iso-Seq) and Illumina RNA sequencing (RNA-seq) technologies to assess the expression of *GSK3β* mRNA and alternative splicing, as well as those related to miRNA, and lncRNA in DSE testes. We analyzed the molecular characteristics of the *GSK3β* gene, and its corresponding protein functions, and conducted protein–protein interaction and correlation analyses. Additionally, we constructed a competitive endogenous RNA (ceRNA) regulatory network by annotating the *GSK3β* gene and identified associated GO terms, miRNAs, and IncRNAs. This study underlined the significance of *GSK3β* in the testis, providing a valuable resource for further study of the mechanisms and functions of the *GSK3β* gene in DSE spermatogenesis.

## 2. Materials and Methods

### 2.1. Short-Read RNA-Seq and Long-Read Iso-Seq

We conducted transcriptomic analysis of *GSK3β* in DSE testes using an integrated approach, combining short-read RNA-seq and long-read Iso-seq. We collected the testicular samples from three 12-month-old mature male DSE. We obtained the short-read sequencing data from Novogene Co., Ltd., Tianjin, China, while long-read sequencing data were obtained from GrandOmics Co., Ltd., Wuhan, China. We merged the unannotated transcripts with annotated transcripts from the Ensembl database to generate a comprehensive annotation file. The filtering and processing of second-generation sequencing data, coupled with the incorporation of newly identified subtypes from third-generation sequencing, facilitated the generation of BAM files. We visualized *GSK3β* gene transcripts using the Sashimi Plot function in IGV. Subsequently, we constructed the pig reference genome (Sus scrofa 11.1) index using STAR-2.5.2 [15]. We calculated gene expression quantification, comprising raw expression levels and normalized expression values (TPM) using FeatureCounts-2.0.1 and Salmon-1.5.1, respectively. Finally, we employed the visualization of the expression abundance of the *GSK3β* gene using R package Gviz.

### 2.2. Transcript Amplification and Sequence Determination

We designed the primers F1/R1 (F1: GCGTTTATCATTAACCTAACACC; R1: ATTTTCTTTCCAAACGTGACC) to amplify the transcript ENSSSCT00000039364 using Premix Taq™ (Takara, Dalian, China). The total reaction volume of 25 μL comprised 12.5 μL of Premix, 1 μL each of 10 μM F1/R1 primers, 1 μL of 50 ng/μL testis cDNA, and the remaining volume was filled with H_2_O. The amplification program consisted of initial denaturation at 95 °C for 5 min, followed by 35 cycles of denaturation at 95 °C for 30 s, annealing at 61 °C for 30 s, extension at 72 °C for 80 s, and a final extension at 72 °C for 10 min.

### 2.3. Characteristics Analysis of Transcript ENSSSCT00000039364

To obtain the complete coding sequence (CDS) of the *GSK3β* transcript ENSSSCT00000039364, we utilized Lasergene 7.1 to analyze the sequencing data. To decipher the feature information of *GSK3β*, we used ProtParam to estimate the molecular weight, molecular formula, and isoelectric point. We parsed the functional domain, secondary structure, tertiary structure, hydrophobic structure, transmembrane helices, and signal peptide of the GSK3β protein using the SMART, SOPMA, I-TASSER, ProtScale, TMHMM2.0, and SignalP6.0 websites, respectively. Finally, we measure the evolutionary relationship of GSK3β amino acid sequences across different species using MEGA11.

### 2.4. Protein–Protein Interaction Analysis of GSK3β

We constructed the protein–protein interaction network using String 11.5. Additionally, for these proteins, we employed GO and KEGG functional enrichment analysis using the R package clusterProfiler. In our analysis, entries with a significance threshold of *p* < 0.05 were considered to be statistically significant. Finally, we associated the identified proteins with gene expression obtained from transcriptome sequencing data and calculated the expression correlation between them.

### 2.5. Regulatory Network Analysis of GSK3β

We utilized the annotation of the UniProt database to acquire insights into the biological processes associated with *GSK3β*, including molecular function and biological processes. To capture miRNAs and lncRNAs regulating *GSK3β*, we analyzed the transcriptome data using miRanda 3.3 and RNAhybrid 2.1.2. Further, we visualized the ceRNA (competing endogenous RNA) transcriptional regulatory network using Cytoscape 3.9.1.

### 2.6. Multi-Tissue Expression Analysis of the GSK3β Gene

We designed fluorescent quantitative primers F2R2 (F2: 5′-ACTTCACCACTCAAGAATTGTCA-3′; R2: CTCCGGCATTAGTATCTGAGG) using *GSK3β* mRNA as a templet, with the housekeeping gene *GAPDH* serving as an internal reference (F3: CCTTCATTGACCTCCACTACATGGT; R3: CCACAACATACGTAGCACCAGCATC). Further, we evaluated the mRNA expression of *GSK3β* across 15 different tissues in DSE. Data analysis followed the relative quantification 2^−ΔΔct^ method [16].

### 2.7. Subcellular Localization Detection of GSK3β

Based on the *Xho*Ⅰ and *Kpn*Ⅰ restriction enzyme cleavage sites of the *GSK3β* gene CDS sequence and the multicloning sites of the pEGFP-C1 green fluorescent protein eukaryotic expression vector, we designed the primers F3/R3 (F4: CTCGAGCTATGTCAGGGCGGCCCAGAA; R4: GGTACCTCAGGTGGAATTGGAAGCTGACG) for the eukaryotic expression of the cDNA sequence. We constructed the pEGFP-C1-*GSK3β* eukaryotic expression recombinant plasmids and transfected them into ST cells to locate the GSK3β and non-transfected ST cells, and those cells transfected with the EGFP-C1 vector served as the negative controls. Subsequently, we stained the nuclear and mitochondria of ST cells using blue Hoechst 33,342 and red MitoTracker, respectively. Finally, we captured the expression and localization of *GSK3β* in ST cells using inverted fluorescent microscopy.

## 3. Results

### 3.1. Alternative Splicing of GSK3β

**Figure 1:**
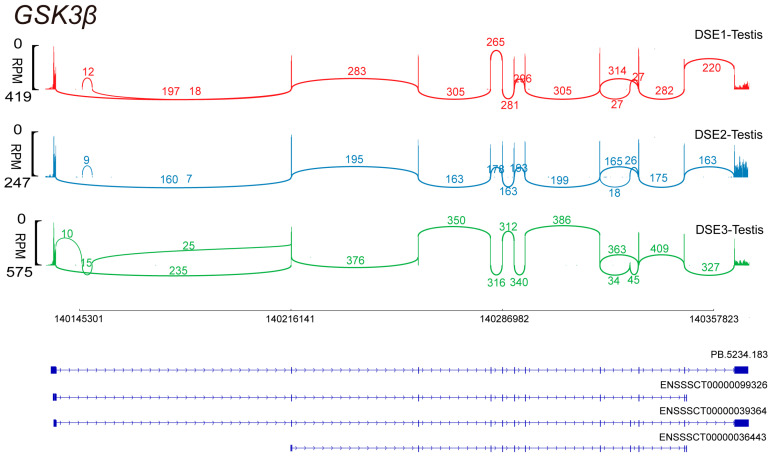
Transcripts and expression levels of *GSK3β* from three testes of DSE pigs.

The short- and long-read sequencing results revealed four distinct alternative splicing isoforms in the testes of DSE pigs. Specifically, novel transcript PB.5234.183 was identified from third-generation sequencing data. Notably, ENSSSCT00000039364 emerged as the predominant transcript, as shown in Figure 1.

### 3.2. GSK3β Gene Expression Characteristics

**Figure 2:**
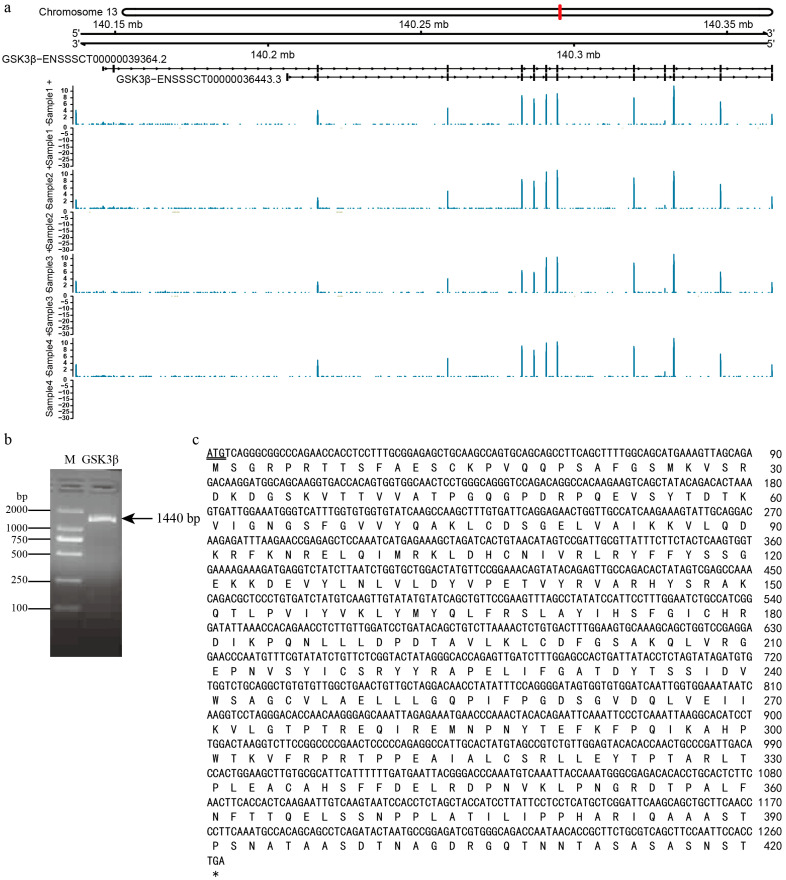
Gene structure of *GSK3β*. (**a**) Chromosome location, exon, intron abundance based on transcriptome sequencing; (**b**) RT-PCR product of DSE *GSK3β* gene; (**c**) *GSK3β* gene coding sequence and amino acid sequence M, DL2000 DNA marker; *GSK3β* PCR product; double underline, start codon; asterisk, stop codon; letters in upper line, nucleic acid sequence; letters in lower line, amino acids sequence.

The transcript ENSSSCT00000039364 of the *GSK3β* gene in DSE pig testes was located on chromosome 13 of the pig genome (Sscrofa11.1), with a total length of 227,644 bp. Gene annotation conducted by Gviz delineated transcript ENSSSCT00000039364, which comprised 12 exons and 11 introns, with consistently high expression across all three DSE pig samples (Figure 2a). A 1440 bp fragment of the *GSK3β* gene was obtained using primers F1/R1 (Figure 2b). Subsequent Sanger sequencing unveiled the full-length coding sequence (CDS) of *GSK3β* as 1263 bp, encoding a total of 420 amino acids (Figure 2c). The GSK3β protein contains five classes of functional active sites: N-glycosylation, proteinase C phosphorylation, casein kinase II phosphorylation, tyrosine kinase phosphorylation, and N-myristoylation.

### 3.3. Homology Analysis of GSK3β Proteins across Species

**Figure 3:**
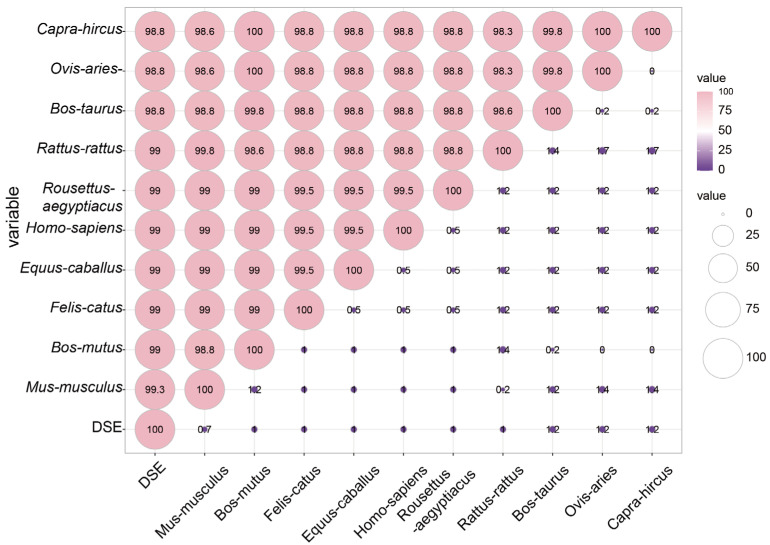
GSK3β proteins homology across different species.

The multiple sequence alignment of GSK3β from various mammalian species revealed a remarkable amino acid sequences similarity exceeding 98.8% among DSE pig, mouse (*Mus musculus* NP_062801), yak (*Bos mutus* XP_005905492), cat (*Felis catus* XP_003991723), horse (*Equus caballus* XP_001502517), human (*Homo sapiens* NP_001139628), bat (*Rousettus aegyptiacus* XP_016014292), rat (*Rattus rattus* XP_032756083), cattle (*Bos taurus* XP_024848670), sheep (*Ovis aries* NP_001123212), and goat (*Capra hircus* AIU41103) (Figure 3), indicating that the GSK3β protein harbored identical functional domains across 11 species.

### 3.4. Protein–Protein Interaction

**Figure 4:**
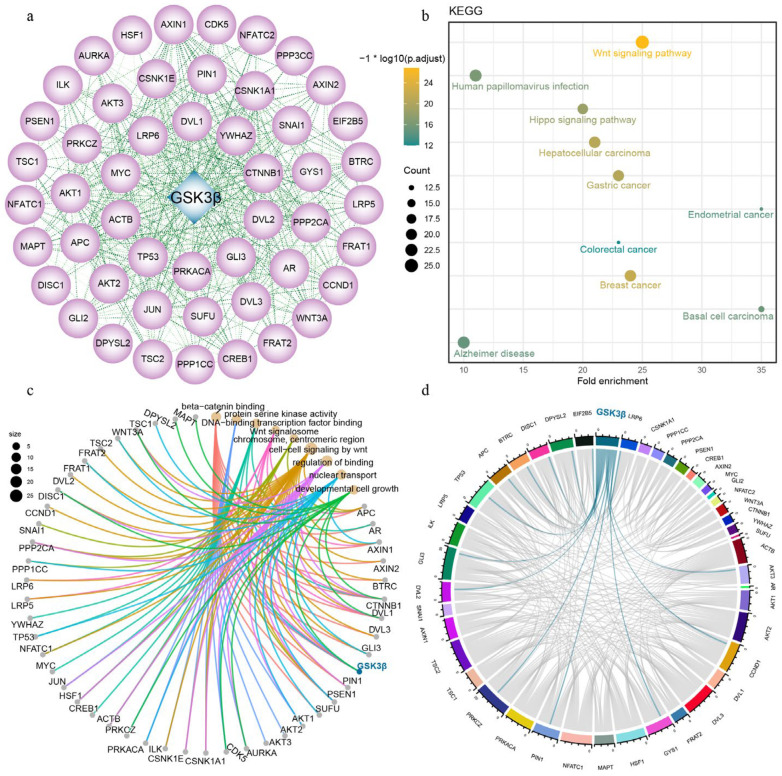
Protein interactive analysis. (**a**) GSK3β protein interaction network; (**b**) KEGG enrichment analysis of interacting proteins; (**c**) GO enrichment analysis of interacting proteins; (**d**) GSK3β correlation chord plot.

The protein–protein interaction analysis revealed 50 proteins that potentially interacted with GSK3β (Figure 4a). The subsequent KEGG enrichment analysis indicated that these proteins are mainly involved in pathways such as the Wnt signaling pathway, human papillomavirus infection, hippo signaling pathway, hepatocellular carcinoma, gastric cancer, colorectal cancer, breast cancer, endometrial cancer, basal cell carcinoma, and Alzheimer’s disease (Figure 4b). Further, GO enrichment analysis indicated that these proteins were primarily involved in β-catenin binding, protein serine kinase activity, DNA-binding transcription factor binding, Wnt signalosome, chromosome, centromeric region, cell–cell signaling by wnt, regulation of binding, nuclear transport, and developmental cell growth (Figure 4c). Finally, we matched these proteins with gene expression data from DSE pigs and identified significant correlations between GSK3β and GYS1, LRP6, PIN1, PRKCZ, TP53, AKT3, APC, BTRC, CCND1, DISC1, DPYSL2, DVL2, EIF2B5, as well as GLI3, with correlation coefficients of 0.99792 to 0.95129, respectively (Figure 4d).

### 3.5. ceRNA Regulatory Network of GSK3β

**Figure 5:**
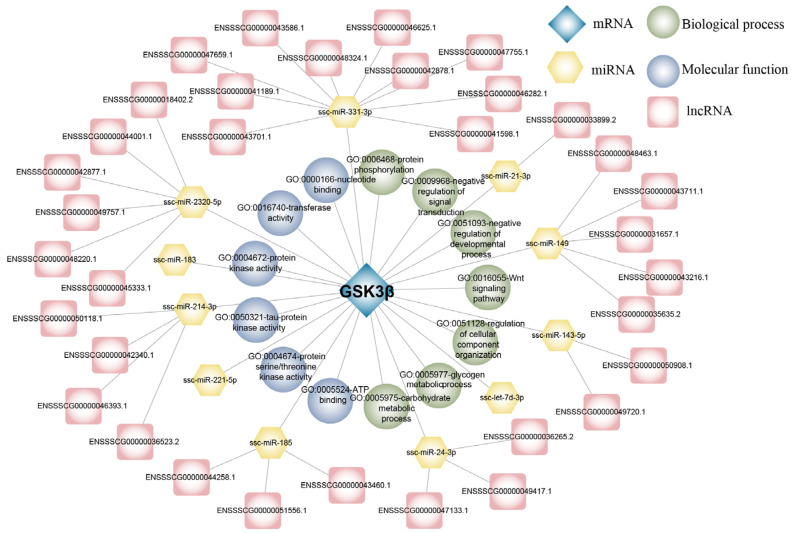
The functional annotation *GSK3β* potential ceRNA regulatory network.

The functional annotation of *GSK3β* revealed its involvement in various biological processes, including protein phosphorylation, negative regulation of signal transduction, negative regulation of developmental process, Wnt signaling pathway, regulation of cellular organization, glycogen metabolic process, and carbohydrate metabolic process. In terms of molecular function, *GSK3β* was primarily involved in nucleotide binding, transferase activity, protein kinase activity, tau protein kinase activity, protein serine/threonine kinase activity, and ATP-binding. Additionally, 11 miRNAs were identified as primary regulators of porcine *GSK3β*, namely ssc-miR-331-3p, ssc-miR-2320-5p, ssc-miR-183, ssc-miR-214-3p, ssc-miR-221-5p, ssc-miR-185, ssc-miR-24-3p, ssc-let-7d-3p, ssc-miR-143-5p, ssc-miR-149, and ssc-miR-21-3p. Among them, ten, six, five, four, three, three, two, and one lncRNA competed with ssc-miR-331-3p, ssc-miR-2320-5p, ssc-miR-149, ssc-miR-214-3p, ssc-miR-185, ssc-miR-24-3p, ssc-miR-143-5p, and ssc-miR-21-3p for binding to GSK3β, respectively (Figure 5).

### 3.6. Expression Pattern of GSK3β across Multi-Tissue

**Figure 6:**
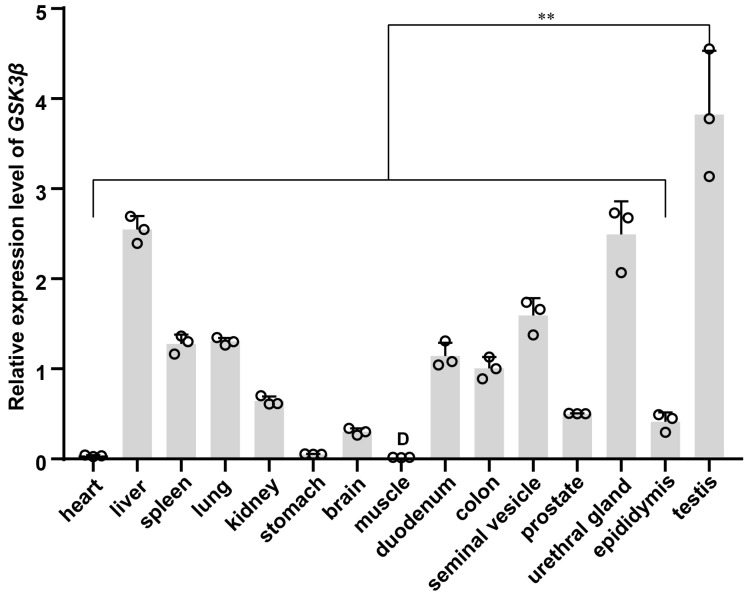
The multi-tissue expression pattern of the *GSK3β* gene. **, highly significant differences (*p* < 0.01).

Multi-tissue qPCR analysis revealed that the relative expression of *GSK3β* displayed the highest level in the testis of DSE pigs. Conversely, relatively low expression levels were observed in the liver, spleen, lung, kidney, brain, duodenum, colon, seminal vesicle, prostate, urethral gland, and epididymis. The expression levels were almost negligible in the heart, stomach, and muscle. The relative expression level of *GSK3β* was significantly higher in testis tissues than in other tissues (*p* < 0.01) (Figure 6).

### 3.7. Subcellular Localization Results of GSK3β

**Figure 7:**
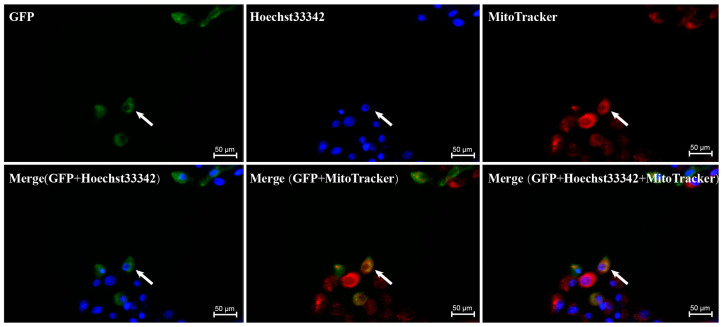
The subcellular localization results of GSK3β protein. Green, blue, and red represent GSK3β, nucleus, and mitochondria, respectively.

The subcellular localization analysis indicated that the majority of the GSK3β protein was located in the cytoplasm of ST cells, with a small amount detected in the nucleus (Figure 7).

## 4. Discussion

In this study, we conducted an in-depth analysis of the *GSK3β* transcriptome in DSE pig testis using a comprehensive approach involving short-read RNA-seq and long-read Iso-seq. Our analysis unveiled four prominently expressed isoforms in DSE pig testis. Notably, ENSSSCT00000039364 emerged as the most significant isoform. Consequently, we proceeded with our further analysis of this specific transcript. The complete coding sequence (CDS) of the *GSK3β* gene was amplified from DSE pig testis cDNA, yielding a sequence length of 1263 bp encoding 420 amino acids. Protein phosphorylation is a fundamental and widely recognized mechanism for regulating and controlling protein activity. GSK3, with multiple phosphorylation sites such as proteinase C and casein kinase II, plays a role in regulating sperm motility and the acrosome reaction via phospho-ser9-GSK3β, contributing to the regulation of sperm energy metabolism [3]. Certain GSK3β substrates necessitate phosphorylation on serine or threonine residues by another protein kinase, utilizing a phosphate group as an identifying element to phosphorylate the substrate [17]. Moreover, *GSK3β* exhibited high conservation across multiple animal species, with over 98.8% similarity observed compared to 10 other animals.

Using GSK3β alone does not guarantee the normal capacitation and acrosome reaction of sperm. It needs to cooperate with other proteins to effectively function in these processes. The analysis of protein–protein interactions unveiled that GSK3β had the potential interaction with 50 different proteins. These proteins were primarily involved in pathways related to the Wnt signaling pathway, human papillomavirus infection, and other processes, according to KEGG enrichment analysis. Furthermore, GO analysis revealed their involvement in various biological processes, including protein serine kinase activity and DNA-binding transcription factor binding. Collectively, these signaling pathways constitute an intricate network regulating both spermatogenesis and the development of cancerous lesions. We also found that GSK3β significantly correlated with several proteins, such as GYS1, and that insulin deficiency can inhibit glycogen synthesis by activating GSK3, which suppresses muscle GYS1 (glycogen synthase 1) and thus maintains the activated form of glycogen phosphorylase [18]. Notably, individuals with type 2 diabetes mellitus (T2DM) experience an elevation in the levels of UDP-glucose (uridine diphosphoglucose), an active intermediate in the testes. Although GYS1 is identified as a hypoxia-inducible factor, the evaluation of testicular GYS1 levels in T2DM rats reveals no significant alterations [19]. The inhibition of GSK3 by Wnt signaling peaks in mitosis, and the Wnt co-receptor LRP6 (low-density lipoprotein receptor-related protein 6) is activated in a cell-cycle-dependent manner by CCNY (cyclin Y) and its target kinase CDK14 (cyclin-dependent kinase 14) [20]. Furthermore, GSK3/LRP6 interactions play a role in gene expression, which could be the foundation of sperm physiology [21]. Prolyl isomerase (PIN1) is essential for promoting the production of spermatozoa through spermatogonial processes in stem cells. Moreover, the maintenance of the blood–testicular barrier depends on the expression of PIN1 in supporting cells [22]. The inhibition of AKT3 expression leads to cell cycle arrest in embryonic stem cells [23]. Studies utilizing apigenin to treat spermatogonia revealed the down-regulation of AKT3 expression, affirming its role in inhibiting spermatogonial stem cell proliferation and promoting abnormal differentiation [24]. Schizophrenia 1 (DISC1) is identified as a risk factor for both schizophrenia and affective disorders. Investigation into the C-terminal structure of mutant DISC1 and an analysis of its expression levels in male and female mice revealed an interruption in the GSK3β signaling pathway, suggesting a significant interaction between these factors [25]. The DVL protein exhibits widespread expression in the body during development. ASPM (abnormal spindle-like microcephaly associated protein) participates in the degradation of DVL2 by counteracting autophagy, consequently activating the Wnt/β-catenin signaling pathway [26]. In summary, the identification of these protein interactions with GSK3β provided valuable insights, paving the way for further research to deepen our understanding of GSK3β’s function and molecular mechanisms in DSE pigs.

MicroRNAs (miRNAs) are short, single-stranded non-coding RNAs typically comprising 19–22 nucleotides, originating from local hairpin structures processed by two RNase III enzymes, Drosha and Dicer. Functionally, miRNAs negatively regulate gene expression post-transcriptionally by binding to complementary sites within the 3′UTR region of target mRNAs [27,28]. These molecules wield significant influence across diverse biological processes, including development, apoptosis, proliferation, differentiation, transformation, and cellular senescence [29,30]. Functional annotation of the porcine *GSK3β* gene and construction of the ceRNA regulatory network revealed that *GSK3β* was targeted by 11 miRNAs. Notably, ssc-miR-331-3p, ssc-miR-2320-5p, ssc-miR-149, and ssc-miR-185 have been the focus of more extensive investigations in this regard. Modulating the expression of ssc-miR-331-3p at various stages of adipocyte development can enhance intramuscular fat deposition without a concurrent increase in subcutaneous fat [31]. Several miRNAs may regulate a single gene, and conversely, a single miRNA can influence multiple genes. Notably, ssc-miR-2320-5p and ssc-miR-149 target TRAF3 (TNF receptor-associated factor 3), a versatile molecule within the TRAF family [31,32]. Furthermore, ssc-miR-2320-5p is implicated in host protective responses and parasite growth [33], while ssc-miR-149 is associated with the regulation of precocious traits in boars. It exhibits high expression levels in immature boars and targets the *SPATA3* gene, which was demonstrated to be the most significantly down-regulated in the testes of infertile patients [34] and plays a pivotal role in mouse spermatogonia development [35]. Additionally, ssc-miR-185 was involved in immune cell differentiation [33]. In summary, miRNAs are central contributors not only in spermatogenesis but also in gene expression, viral infection, and cancer. LncRNA is a crucial contributor to various biological processes and has emerged as a significant regulator in diverse biological environments. According to GO analysis, the principal enrichment items are associated with a range of physiological response processes, including protein phosphorylation, and the negative regulation of signal transduction, indicating the pivotal role of the *GSK3β* gene in diverse physiological processes.

*GSK3β* is predominantly found in the central nervous system, often localized in axons, and it serves as the primary kinase responsible for phosphorylating tau proteins. The overexpression or overactivity of *GSK3β* increases tau protein phosphorylation, which in turn leads to altered axonal transport and hippocampal neurodegeneration [36] and learning disabilities [37]. Notably, *GSK3β* is expressed in goat sperm, particularly around the acrosome, in the middle and along the main tail, with its abundance increasing during the epididymal transport process [3]. Multi-tissue qPCR analysis revealed that *GSK3β* was universally expressed across various tissues in DSE pigs, with high expression levels in the testis, underscoring its multifunctionality and significant role within organisms. At the cellular level, our subcellular localization findings revealed predominantly cytoplasmic expression in ST cell lines.

## 5. Conclusions

The integration of short-read and long-read sequencing methodologies provided a comprehensive insight into the transcriptional regulation and expression of *GSK3β*. Through an investigation into the transcriptional complexity arising from alternative splicing events within *GSK3β*, transcript characteristics, ceRNA-mediated regulatory, and the expression profiles at both mRNA and cellular levels were elucidated. Notably, four distinct transcripts were identified. ENSSSCT00000039364 exhibited 12 exons and displayed a conserved amino acid sequence across 11 species. Additionally, 50 proteins interacting with *GSK3β* were delineated, mainly involving the Wnt signaling pathway, multiple cancers, and Alzheimer’s disease. Significant associations were observed between GSK3β and GYS1, LRP6, PIN1, PRKCZ, TP53, AKT3, APC, BTRC, CCND1, DISC1, DPYSL2, DVL2, EIF2B5, as well as GLI3. *GSK3β* was primarily involved in thirteen GOs, encompassing six molecular functions and seven biological processes. Eleven miRNAs were identified as regulators of *GSK3β* expression. The substantial expression of *GSK3β* was detected in the testes, predominantly localized within the cytoplasm of ST cells. These findings broaden our understanding of the transcriptional regulatory properties underlying the spermatogenesis-related gene *GSK3β*, thereby laying the foundation for further elucidation of its function and molecular mechanism within the testes of DSE pigs, particularly in the context of sperm capacitation and acrosome reaction.

## Data Availability

The sequencing data were deposited in the NCBI Gene Expression Omnibus under the accession number GSE230506.
